# Anthelmintic Resistance in *Ancylostoma caninum*: A Comprehensive Review

**DOI:** 10.1002/vms3.70434

**Published:** 2025-05-28

**Authors:** Hande İrem Sönmez, Elif Madak, Mina Cansu Karaer, Hıfsı Oğuz Sarımehmetoğlu

**Affiliations:** ^1^ Graduate School of Health Science, Ankara University Ankara Türkiye; ^2^ Department of Parasitology Faculty of Veterinary Medicine Ankara University Ankara Türkiye; ^3^ Institute of Preclinical Sciences Veterinary Faculty University of Ljubljana Ljubljana Slovenia

**Keywords:** *Ancylostoma caninum*, anthelmintic resistance, hookworms, veterinary parasitology, zoonotic infections

## Abstract

*Ancylostoma caninum*, a zoonotic hookworm species, significantly affects the global health of companion animals, humans and wildlife populations. This parasitic infection is prevalent in various environments, particularly in regions with warm climates, and affects a wide range of canids, including dogs, where it is most commonly found. *A. caninum* is a major concern not only due to its zoonotic potential but also because of its growing resistance to anthelmintic treatments. The development of resistance in parasitic species is primarily driven by genetic mutations that allow the parasite to survive treatment with commonly used drugs and presents a serious challenge to parasite control efforts. This review explores the biology and epidemiology of *A. caninum*, focusing on the mechanisms and prevalence of anthelmintic resistance. By reviewing worldwide studies, this paper highlights the prevalence of resistance across different anthelmintic classes and its implications for veterinary and public health. The findings emphasize the need for better management strategies and innovative solutions to address this growing problem.

## Graphical abstract

This review summarizes the emergence of anthelmintic resistance in *Ancylostoma caninum*, with a focus on drug classes affected, global prevalence and resistance mechanisms. Early detection and integrated control strategies are essential to combat its spread.

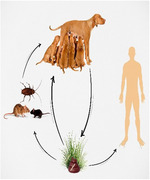

## 1 Introduction

*Ancylostoma caninum*, which belongs to the Ancylostomatidae family and has zoonotic significance, is a harmful hookworm found in domestic dogs and wild canids, rarely in felids (Liu et al. 2013), named for the characteristic dorsal curve of its buccal capsule, which resembles a ‘hook’ (Bowman 2021). The pre‐adult and adult stages of A. caninum reside in the small intestine of their canine hosts. The nematode's life cycle is direct, although paratenic hosts can occasionally play a role (Little 1961, Figure 1).

**FIGURE 1:**
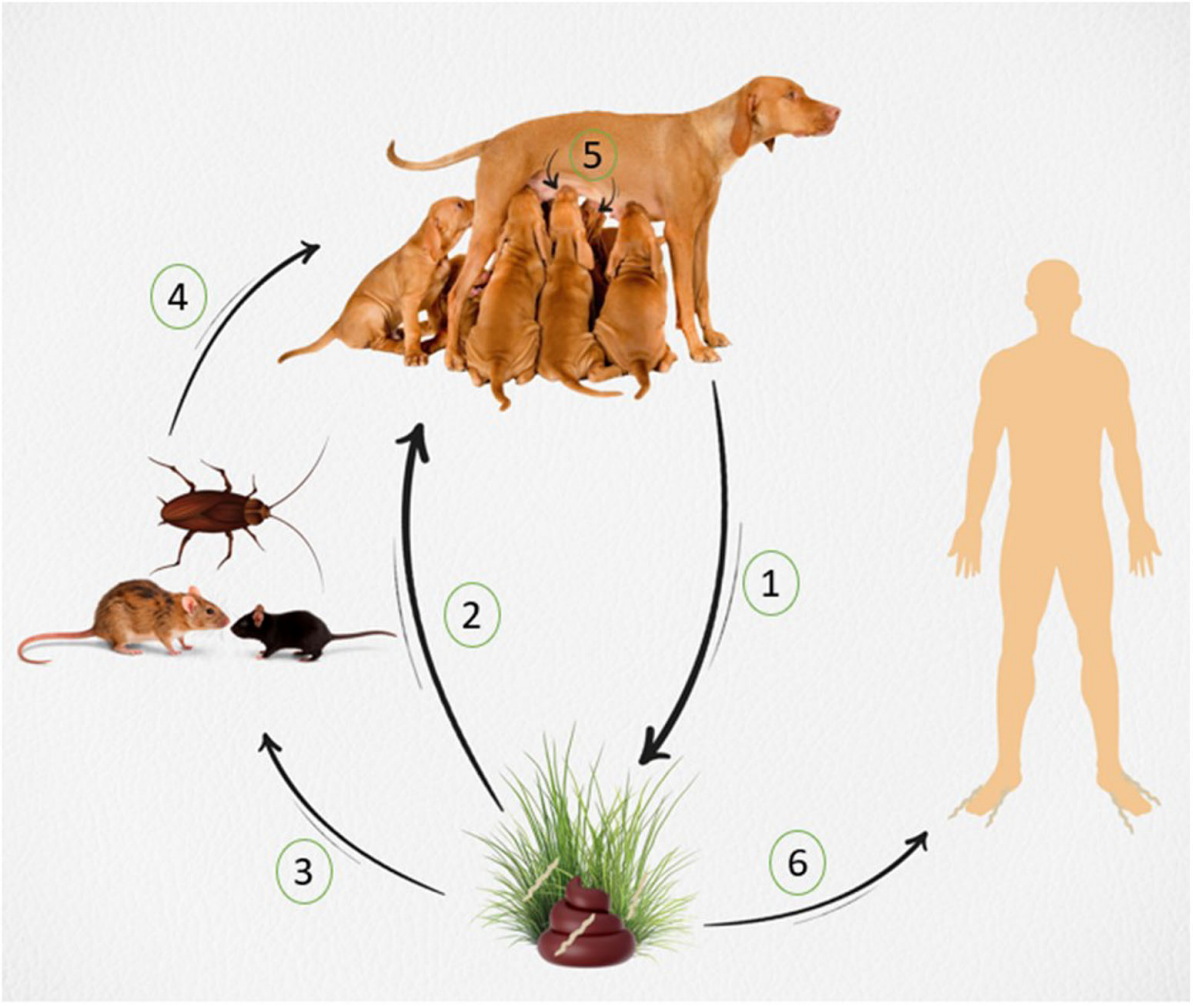
Life cycle of *Ancylostoma caninum*, illustrating key transmission routes. (1) Eggs are excreted in the faeces, from which L1 larvae hatch and develop into infective L3 larvae. (2) Dogs can become infected by ingesting L3 larvae through the oral route. 3.L3 larvae can also be consumed by paratenic hosts, where they remain infective. (4) When dogs ingest paratenic hosts harbouring L3 larvae, the infection is transmitted. (5) Transmammary transmission can occur, allowing larvae to pass from the mother to her offspring. (6) In humans, infective L3 larvae can penetrate the skin, leading to cutaneous larva migrans.

Infection arises when infective third‐stage larvae (L3) are ingested orally or penetrate the skin. Eggs expelled in faeces contain first‐stage larvae, which hatch and develop into second‐stage larvae. The second‐stage larvae then mature into infective third‐stage larvae in 2–8 days at 23°C–30°C (Güralp 1981; Soulsby 1965).

Infection can be transmitted via skin, oral, transmammary, and paratenic host routes (Taylor et al. 2016; Epe 2009). In skin infections, larvae penetrate the skin, enter the bloodstream and lymphatic system to the right atrium, and then make their way to the lungs. Many of these larvae attach to the capillaries of the lungs and pass into the alveoli. From the alveoli, the larvae move up the trachea and pharynx, are swallowed, and then travel via the oesophagus to the small intestine, where they develop into adults. During this migration, the larvae continue to feed but do not develop further (Hawdon et al. 1993). Some larvae migrate to the muscles and intestinal walls, where they enter an inhibited state during the third‐stage larva period (Hawdon and Wise 2021). Because of pregnancy, larvae that were inhibited prior to birth become active, travel to the mammary glands shortly before delivery, and are excreted in the milk. Puppies that suckle the milk ingest these larvae and become infected via the transmammary route (Miller 1981; Stone and Girardeau 1968; Taylor et al. 2016). For parasites that enter the host through the skin and mature in the intestine without entering inhibition, the prepatent period in puppies is approximately 14–17 days. In older dogs, this period can extend up to 26 days (Doğanay and Yıldız 2018).

Most of the larvae ingested orally directly enter the gastrointestinal system, without migrating to the lungs. The infective larvae remain within the intestinal mucosa for the first few days before returning to the intestinal lumen. The prepatent period is approximately 15–26 days. A small number of larvae, however, penetrate the oral cavity, pharynx, or intestinal wall, entering the venous system. These larvae may undergo pulmonary migration before returning to the gastrointestinal system or migrate to other organs and enter an inhibited state. Infective larvae activated by pregnancy contribute to transmammary transmission (Güralp 1981; Soulsby 1965). Infection can also occur through the ingestion of paratenic hosts, such as mice and rats, that harbour third‐stage larvae (Daba et al. 2021). In addition, *A. caninum* third‐stage larvae can invade the muscles of cockroaches, where they can remain viable for extended periods (Doğanay and Yıldız 2018; Little 1961).

One of the fascinating biological characteristics of *A. caninum* infection is the phenomenon known as ‘larval leak’. Somatic larvae in the tissues continuously migrate to the small intestine, where they develop into adult worms (Epe 2009). This process occurs independently of pregnancy. In dogs experiencing this phenomenon, hookworm eggs are chronically observed in faeces (Bowman 2021; Jimenez Castro et al. 2019). Due to hypobiotic larvae that reactivate in the intestine after treatment and initiate a new egg‐shedding cycle, egg detection in faeces only ceases temporarily (Jimenez Castro et al. 2019).

The pathogenicity of hookworms varies depending on the host's resistance and the parasite burden, ranging from asymptomatic infections to severe, life‐threatening blood loss (Bowman 2021). The treatment and control of this infection primarily rely on anthelmintic drugs. However, improper or excessive drug use contributes to resistance development in parasite populations, reducing treatment efficacy and increasing the risk of new infections. When an anthelmintic is used at an appropriate dose, it initially affects a significant portion of the parasite population. Over time, however, genetic resistance may develop, leading to inherited tolerance within the population. This phenomenon is known as anthelmintic resistance (Prichard et al. 1980). As previously mentioned, *A. caninum* is a zoonotic parasite (Prociv and Croese 1996), and its treatment and control have significant implications for both animal (Traversa 2012; Bowman 2021) and human health (Landmann and Prociv 2003; Mahdy et al. 2012).

The aim of this review is to comprehensively examine the mechanisms, prevalence, and impact of anthelmintic resistance in *A. caninum*. Given the serious implications of anthelmintic resistance for both veterinary medicine and public health, understanding this issue from a ‘One Health’ perspective is crucial, considering the interconnected nature of human, animal, and environmental health.

## 2 Review Methodology

We searched the following databases: PubMed, Web of Science, Google Scholar and Scopus (keyword search terms used: ‘*A. caninum*’, ‘anthelmintic resistance’, ‘drug resistance’, ‘prevalence’ and ‘epidemiology’). In addition, references cited in the retrieved articles were reviewed to identify further relevant studies.

## 3 Epidemiology and Spread

*A. caninum* is a zoonotic parasite with significant public health implications. While it primarily parasitizes dogs, it can also infect felids, wild carnivores and humans. In humans, *A. caninum* can cause cutaneous larva migrans (CLMs) and is more commonly found in warm and humid climates (Taylor et al. 2016). It is particularly prevalent in subtropical and temperate regions, with infections most frequently reported in dogs under 1 year of age in endemic areas. In older animals, clinical disease is less common due to acquired immunity (Taylor et al. 2016).

Infection with *A. caninum* can occur through various transmission routes, including faecal–oral ingestion of eggs from infected dog faeces, transmammary transmission and skin penetration by hatched larvae (Epe 2009). Under favourable environmental conditions, larvae can serve as an infection source for both humans and animals. While infective larvae can survive in moist soil, they are not resistant to drought (Soulsby 1965). Environmental contamination often results from dogs being walked on grass or dirt paths, where moisture helps retain larvae while protecting them from sunlight. On such surfaces, larvae can survive for several weeks. In addition, the housing conditions of dogs play a crucial role. Kennels with moist, porous or cracked flooring can lead to significant accumulation of infectious larvae (Taylor et al. 2016).

Hygiene conditions, dog population density and climatic factors play a critical role in the spread of the parasite. Poor sanitation and environmental contamination with infected faeces allow larvae to survive in the soil, while warm and humid climates further support the parasite's development (Strunz et al. 2014). Regular anthelmintic treatments, environmental sanitation and awareness campaigns are among the most effective strategies for controlling the spread of *A. caninum*.

## 4 Mechanism of Action of Anthelmintics and Resistance Development

Two centuries ago, parasite treatments used rudimentary methods like metals and plant extracts (Lees et al. 2022). By the 20th century, arsenicals and nicotine sulphate offered limited efficacy. Between 1960 and 1980, drugs like thiabendazole and levamisole marked significant progress (Kates et al. 1971; McKellar and Jackson 2004). The 1981 introduction of ivermectin revolutionized treatment but led to rapid resistance, prompting research into anthelmintic mechanisms (Shoop and Soll 2002; Mukherjee et al. 2023).

Understanding anthelmintic resistance requires first comprehending the mechanisms of action of active compounds. Knowledge of these mechanisms is crucial for explaining how anthelmintics work and how resistance develops. Benzimidazole group drugs are among the most used anthelmintics due to their low cost, broad‐spectrum efficacy, and ease of use. However, their high affinity for β‐tubulin, a key component of the cytoskeleton involved in microtubule formation, represents a major resistance issue for this drug class (Furtado et al. 2016).

Benzimidazole anthelmintics disrupt the structural integrity of microtubules by preventing the formation of α+β tubulin dimers. They selectively bind to the parasite's β‐tubulin with high affinity, inhibiting microtubule polymerization. This leads to the destruction of cellular structures and ultimately results in parasite death (Geary 2016; Abongwa et al. 2017).

Macrocyclic lactones act as selective agonists of glutamate‐gated chloride channels (GluCls), which are present in the neurones and pharyngeal muscles of nematodes and arthropods. The activation of GluCls by macrocyclic lactones inhibits movement and pharyngeal pumping. Consequently, in certain species, paralysis of somatic and pharyngeal muscles leads to either the expulsion of the parasite or its starvation (Geary 2016; Abongwa et al. 2017).

Tetrahydropyrimidines are classified as nicotinic agonists. They act as acetylcholinesterase inhibitors within the nervous system of nematodes. By disrupting nerve signal transmission, in which acetylcholine functions as a neurotransmitter, they cause parasite paralysis and prevent attachment to the intestinal wall (Vardanyan and Hruby 2016; Abongwa et al. 2017).

Anthelmintic resistance in a parasite population can be inherited through one or more resistance‐associated genes (Fissiha and Kinde 2021, Figure 2). As a result, resistance can emerge even when a drug is used for the first time (Silvestre and Humbert 2002; Shalaby 2013). When an anthelmintic is administered at an appropriate dose, any parasites that survive are those carrying resistance‐conferring genes (Shalaby 2013).

**FIGURE 2:**
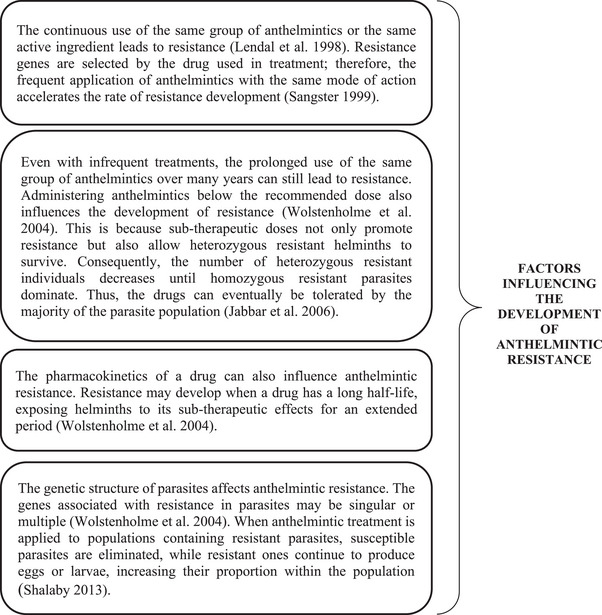
Key factors contributing to developing anthelmintic resistance, including genetic, pharmacokinetic and treatment‐related aspects.

Anthelmintic resistance in helminths is a recessive trait. Consequently, only homozygous helminths can tolerate an appropriate anthelmintic dose, whereas heterozygous parasites are eliminated by the drug (Shalaby 2013). The parasite's direct or indirect development affects resistance. Helminths with a direct life cycle can transmit resistance‐associated alleles directly to the next generation (Wolstenholme et al. 2004).

## 5 Anthelmintic Resistance in *A. caninum*

For the treatment of *A. caninum*, febantel and fenbendazole from the benzimidazole class, moxidectin and milbemycin oxime from the macrocyclic lactone class, and pyrantel from the tetrahydropyrimidine class are commonly used. Studies have reported that febantel, moxidectin and milbemycin oxime exhibit over 99% efficacy against *A. caninum*, while fenbendazole shows more than 98% efficacy. Pyrantel, on the other hand, has demonstrated an average efficacy of approximately 94% across various studies (Jimenez Castro et al. 2020). Although these drugs have been found to be highly effective, in recent years, cases of *A. caninum* infections that do not respond to treatment have been increasingly reported. In this context, between 1987 and 2024, a total of 23 studies investigating anthelmintic resistance in *A. caninum* have been conducted in Australia, the USA, Brazil, Canada and New Zealand (Table 1).

**TABLE 1:** Summary of published studies on anthelmintic resistance in *A. caninum*.

Year	Country	Anthelmintic resistance detection method	Resistant drug	Reference
1987	Australia	Critical trial	Pyrantel pamoate Oxantel pamoate	Jackson et al. 1987
1991	Australia	Critical trial	Pyrantel embonate	Hopkins and Gyr 1991
1998	Australia	Critical trial	Pyrantel–oxantel–praziquantel	Hopkins et al. 1998
2007	Australia	Controlled trial	Pyrantel embonate	Kopp et al. 2007
2008	Australia	Critical trial Larval motility assay Larval migration assay Larval feeding inhibition assay	Pyrantel embonate	Kopp et al. 2008
2009	USA	Allele‐specific real‐time PCR	Benzimidazole* *(β‐tubulin isotype‐1 codon 198 SNP)	Schwenkenbecher and Kaplan 2009
2014	Brazil	Amplification refractory mutation system (ARMS‐PCR)	Benzimidazole* *(β‐tubulin isotype‐1 codon 200 SNP)	Furtado et al. 2014
2019	USA	Larval development assay (LDA) Allele‐specific real‐time PCR	Ivermectin, thiabendazole, benzimidazole* *(β‐tubulin isotype‐1 codon 167 SNP)	Kitchen et al. 2019
2019	USA	Egg hatch assays (EHA) Larval development assays (LDA) Deep amplicon sequencing assay FECRT	Ivermectin, pyrantel, benzimidazole* *(β‐tubulin isotype‐1 codon 167 SNP)	Jimenez Castro et al. 2019
2020	USA	FECRT	Pyrantel pamoate, fenbendazole, milbemycin oxim	Jimenez Castro et al. 2020
2021	USA	Egg hatch assay (EHA) Larval development assay (LDA) FECRT Deep amplicon sequencing assay	Macrocyclic lactones, benzimidazole* *(β‐tubulin isotype‐1 codon 167 SNP)	Jimenez Castro et al. 2021
2022	Brazil	FECRT	Pyrantel pamoate, praziquantel. fenbendazole	D'ambroso Fernandes et al. 2022
2022	USA	FECRT	Pyrantel pamoate, fenbendazole, milbemycin oxime, moxidectin	Jimenez Castro et al. 2022
2023	USA	Deep amplicon sequencing assay	Benzimidazole* *(β‐tubulin isotype‐1 codon 134 and 167 SNP	Venkatesan et al. 2023
2023	Canada	Allele‐specific real‐time PCR	Benzimidazole* *(β‐tubulin isotype‐1 codon 167 SNP)	Evason et al. 2023
2023	USA	FECRT	Moxidectin‐ imidacloprid, pyrantel pamoate‐febantel‐praziquantel	Balk et al. 2023
2023	USA	Allele‐specific real‐time PCR	Benzimidazole* *(β‐tubulin isotype‐1 codon 167 SNP)	Leutenegger et al. 2023
2023	Australia, New Zealand	Deep amplicon sequencing assay	Benzimidazole* *(β‐tubulin isotype‐1 codon 134,167,200,198 SNP)	Stocker et al. 2023
2024	Canada, USA	FECRT Deep amplicon sequencing assay	Benzimidazole* *(β‐tubulin isotype‐1 codon 167 SNP)	Nezami et al. 2024
2024	USA	Larval development assay (LDA) Egg hatch assay (EHA) FECRT Allele‐specific real‐time PCR Larval activation assay	Thiabendazole, ivermectin, moxidectin, pyrantel pamoate, benzimidazole* *(β‐tubulin isotype‐1 codon 134, 167 SNP)	McKean et al. 2024
2024	USA, Canada	Allele‐specific real‐time PCR	Benzimidazole* *(β‐tubulin isotype‐1 codon 167 SNP)	Leutenegger et al. 2024
2024	USA	Allele‐specific real‐time PCR	Benzimidazole* *(β‐tubulin isotype‐1 codon 167 SNP)	Evason et al. 2024
2024	Australia	FECRT	Pyrantel embonate, oxantel embonate, praziquantel	Dale et al. 2024
2024	Australia, New Zealand	Deep amplicon sequencing assay	Benzimidazole* *(β‐tubulin isotype‐1 codon 134, 167 SNP)	Abdullah et al. 2024

* Indicates benzimidazole resistance as reported in each study, associated with SNPs in the β‐tubulin isotype‐1 gene (e.g., codons 134, 167, 198 or 200, depending on the study).

### 5.1 Australia

Anthelmintic resistance in *A. caninum* was first reported in 1987 in Australia against pyrantel pamoate and oxantel pamoate (Jackson et al. 1987). Subsequently, Hopkins and Gyr (1991) found that pyrantel embonate alone exhibited only 75.1% efficacy against adult *A. caninum*, while Hopkins et al. (1998) reported that the pyrantel–oxantel–praziquantel combination had an efficacy rate ranging from 63.4% to 76%. Studies conducted by Kopp et al. (2008, 2007) confirmed the presence of *A. caninum* isolates resistant to pyrantel embonate. Stocker et al. (2023) analysed β‐tubulin mutations associated with benzimidazole resistance and identified a rare E198K mutation in Australia. Dale et al. (2024) demonstrated that a drug containing pyrantel, oxantel embonate and praziquantel was ineffective in reducing faecal egg counts in Australian dogs. One of the most recent studies by Abdullah et al. (2024) examined mutations in the beta‐tubulin isotype‐1 gene and identified resistance alleles at codon 167 and codon 134.

### 5.2 USA

The first case of benzimidazole resistance in *A. caninum* in the USA was identified by Schwenkenbecher and Kaplan (2009), who developed a real‐time PCR method to detect resistance alleles at codon 198 of the beta‐tubulin isotype‐1 gene. Kitchen et al. (2019) reported an isolate from Florida resistant to both benzimidazoles and ivermectin. Extensive studies by Jimenez Castro et al. (2019, 2020, 2022, 2021) examined *A. caninum* resistance levels across the USA, highlighting the widespread prevalence of the β‐tubulin isotype‐1 F167Y mutation. These studies identified isolates resistant to various anthelmintics, including ivermectin, pyrantel, fenbendazole, milbemycin oxime and moxidectin. Venkatesan et al. (2023) confirmed the association of benzimidazole resistance not only with the F167Y (TTC > TAC) mutation but also with the Q134H (CAA > CAT) mutation. Balk et al. (2023) found that the moxidectin/imidacloprid and pyrantel pamoate/febantel/praziquantel combinations were ineffective against hookworms. Leutenegger et al. (2023) further confirmed the presence of the β‐tubulin isotype‐1 F167Y (TTC > TAC) mutation. McKean et al. (2024) characterized an isolate susceptible to thiabendazole, ivermectin, moxidectin and pyrantel pamoate. Evason et al. (2024) reported an *A. caninum* infection in a greyhound with chronic diarrhoea, confirming the F167Y mutation and observing treatment failure with anthelmintics.

### 5.3 Brazil

In Brazil, Furtado et al. (2014) investigated single nucleotide polymorphisms (SNPs) at codons 198 and 200 in *A. caninum* obtained from two naturally infected dogs. They found an SNP at codon 200 with a low frequency of 0.8%, but no SNP at codon 198. D'ambroso Fernandes et al. (2022) evaluated the efficacy of pyrantel pamoate and praziquantel combination, as well as fenbendazole, in dogs infected with *A. caninum*. They reported a 75% efficacy for each treatment, while the combination of milbemycin oxime and praziquantel showed 100% efficacy.

### 5.4 Canada

In a study by Evason et al. (2023), real‐time PCR was used to detect the benzimidazole resistance polymorphism at codon F167Y of the β‐tubulin isotype‐1 gene. Nezami et al. (2024) conducted research on dog faeces from Canada and the USA to determine benzimidazole resistance. In the US group, they identified the benzimidazole resistance polymorphism at codon F167Y of the β‐tubulin isotype‐1 gene. Similarly, Leutenegger et al. (2024), in collaboration with the US, also identified the benzimidazole resistance at the same codon. In addition, in samples where *Ancylostoma* spp. was detected, they found the highest prevalence of the F167Y polymorphism in breeds including poodles (28.9%), Bernese mountain dogs (25%), Cocker Spaniels (23.1%) and Greyhounds (22.4%).

## 6 Anthelmintic Resistance: Impacts, Management and Control

During the literature review conducted for this study, it has been found that there is an increasing anthelmintic resistance in *A. caninum*, which poses a significant threat to the health of both domestic dogs and wild carnivores, as well as a risk for zoonotic infections (Evason et al. 2023). Besides humans and domestic animals, more than 10 different wild animal species can also become infected with *A. caninum* (Seguel and Gottdenker 2017). Infections have been detected in animals such as coyotes (*Canis latrans*) (Liccioli et al. 2012), red foxes (*Vulpes vulpes*) (Ubelaker et al. 2013), grey wolves (*Canis lupus*) (Guberti et al. 1993), dingoes (*Canis lupus dingo*) (Smout et al. 2013), golden jackals (*Canis aureus*) (Lahmar et al. 2014), grey foxes (*Urocyon cinereoargenteus*) (Conti 1984), bobcats (*Lynx rufus*) (Hiestand et al. 2014) and black bears (*Ursus americanus*) (Foster et al. 2004). In many cases, hookworms can complete their life cycles in wild animals and negatively affect their health. This highlights the importance of the domestic animal–human–wildlife interaction in this disease and the significant role hookworm infections can play in the continuity of the disease (Seguel and Gottdenker 2017).

In cases of *A. caninum* infections in dogs that do not respond to anthelmintic treatment, anaemia and cachexia can be prominent symptoms (Taylor et al. 2016). *A. caninum* also causes a highly pruritic skin infection known as follicular CLMs in humans exposed to infective larvae. The larvae, which enter through the skin, form tunnels in the epidermis for weeks or months but cannot complete their development in humans (Bowman et al. 2010; Hawdon and Wise 2021). A serious infectious eye disease called diffuse unilateral subacute neuroretinitis (DUSN) may occur when infective *A. caninum* larvae migrate to the eyes, leading to vision impairment and blindness. The disease begins with vision loss in one eye, vitreous inflammation and focal pigment epithelial loss and can progress to retinal vascular stenosis, optic atrophy and permanent vision loss (Hawdon and Wise 2021).

Another zoonotic disease caused by hookworms is eosinophilic enteritis, which results from a single mature or immature hookworm settling in the intestines. Severe eosinophilic enteritis is thought to be a Type 1 hypersensitivity reaction triggered by exposure to hookworms in the intestines. Contaminated water or food is considered the most likely route for oral entry of the L3 larvae (Hawdon and Wise 2021).

Effectively managing anthelmintic resistance in *A. caninum* requires a variety of strategic approaches. First, the strategic use of current anthelmintic drugs is critical. Instead of using a single drug, it is necessary to use combined drugs with different mechanisms of action (Marsh and Lakritz 2023). For combined anthelmintic treatment, it has been observed that administering moxidectin (2.5–4.0 mg/kg), pyrantel (5.23–8.64 mg/kg) and febantel (26.17–43.24 mg/kg) on a monthly basis for a duration of 3 to 4 months in dogs effectively inhibits egg shedding (Hess et al. 2019). Regular monitoring of resistance patterns is also an important step. This monitoring allows the early identification of resistance emergence and permits the implementation of targeted interventions.

## 7 Conclusion and Future Perspectives

This review, based on literature surveys on *A. caninum* hookworm, addresses globally developing resistant strains of the parasite and the resistance mechanisms these strains exhibit against anthelmintic drugs. Research indicates that *A. caninum* is a prevalent intestinal parasite, especially among domestic dogs, in various geographical regions. Over time, strains of *A. caninum* resistant to different anthelmintic drugs have been identified, complicating the treatment process and making parasitic disease control more challenging.

Recent studies have shown that *A. caninum* rapidly develops resistance to certain drugs, leading to limited treatment options. Resistance to traditional anthelmintic drugs, particularly benzimidazoles, is reducing global treatment success and causing significant health system issues. However, most of these studies have been region‐specific, with a notable lack of comprehensive research on resistance development in large continents like Europe and Asia. This gap hinders a full understanding of resistance mechanisms on a global scale.

Future research should focus on a deeper examination of the genetic and biological mechanisms behind resistance development in *A. caninum*. The use of molecular biology techniques and the tracking of resistant strain spread could facilitate the development of more effective treatment strategies. Furthermore, the development of alternative therapies and drugs could play a key role in combating these resistant strains. Targeting different stages of the parasite's life cycle could help create more specific treatment approaches. Another crucial aspect is the monitoring of resistance development and the continuous analysis of available data. This process not only contributes to updating treatment strategies but is also vital for both animal and public health. As part of the One Health approach, it is essential to address human, animal and environmental health in an integrated way and develop a long‐term, sustainable strategy for managing resistant *A. caninum* strains.

In conclusion, the global spread of resistant *A. caninum* strains represents a threat not only to animal health but also to human health and ecosystem stability. Therefore, international cooperation and interdisciplinary efforts are necessary to develop effective management strategies and resistance control programmes. These efforts will be essential in overcoming this issue in the future.

## Author Contributions

**Hande İrem Sönmez**: conceptualization, visualization, methodology, writing – original draft, review and editing. **Elif Madak**: writing – review and editing, formal analysis. **Mina Cansu Karaer**: writing – review and editing, formal analysis. **Hıfsı Oğuz Sarımehmetoğlu**: supervision, writing – review and editing, critical manuscript revision. All authors have read and approved the final version of the manuscript.

## Conflicts of Interest

The authors declare no conflicts of interest.

### Peer Review

The peer review history for this article is available at https://publons.com/publon/10.1002/vms3.70434.

## Data Availability

This is a review article, and no new data were generated or analysed in this study.
